# Benefits of skin biopsy of senile hemangioma in intravascular large B-cell lymphoma: A case report and review of the literature

**DOI:** 10.3892/ol.2014.2017

**Published:** 2014-03-31

**Authors:** YASUSHI ADACHI, KOKI KOSAMI, NORITOSHI MIZUTA, MITSUHIRO ITO, YUKI MATSUOKA, MAMI KANATA, HAJIME AKIYAMA, TOMOKO MURAO, MING LI, RYUJI IEKI, SUSUMU IKEHARA

**Affiliations:** 1 Division of Surgical Pathology, Toyooka Hospital, Toyooka City, Hyogo 668-8501, Japan; 2 Department of Stem Cell Disorders, Kansai Medical University, Hirakata City, Osaka 573-1010, Japan; 3 Department of Internal General Medicine, Toyooka Hospital, Toyooka City, Hyogo 668-8501, Japan; 4 Division of Medical Biophysics, Kobe University Graduate School of Health Sciences, Kobe City, Hyogo 650-0017, Japan; 5 Department of Internal Medicine, Public Muraoka Hospital, Kami-Cho, Hyogo 667-1311, Japan; 6 Department of Dermatology, Toyooka Hospital, Toyooka City, Hyogo 668-8501, Japan; 7 Department of Clinical Laboratory, Toyooka Hospital, Toyooka City, Hyogo 668-8501, Japan

**Keywords:** intravascular large B-cell lymphoma, senile hemangioma, skin biopsy, CD20

## Abstract

Intravascular large B-cell lymphoma (IVLBCL) is a rare subtype of B-cell lymphoma characterized by selective growth of clonal B-cells in the lumen of the small vessels of various organs including the liver, spleen, lungs, skin, brain, and kidney. An 86-year-old male presented with weight loss, fever and night sweats (known as B symptoms). Blood examination revealed pancytopenia, high lactate dehydrogenase and high soluble interleukin-2 receptor, suggesting hematopoietic malignancy. However, there were no abnormal hematopoietic cells in the peripheral blood. No lymph node swelling was identified on examination by whole-body computed tomography scan. Therefore, IVLBCL was suspected, and random skin biopsies and a skin biopsy from a senile hemangioma were carried out. A small number of large atypical lymphoid cells resided in the small blood vessels in the deep dermis and subcutaneous tissue of the random skin biopsies, and numerous atypical lymphoid cells were identified in the small vessels of the senile hemangioma. These results suggest the usefulness of skin biopsy from senile hemangiomas in the diagnosis of IVLBCL.

## Introduction

Intravascular large B-cell lymphoma (IVLBCL) is a rare subtype of extranodal B-cell lymphoma defined by intravascular preferential growth of clonal B-cells (1,2). Since Pfleger and Tappeiner reported the first case in 1959 (3), >300 cases have been reported (4). IVLBCL usually occurs in elderly patients and tumor cells affect various organs. There are several systemic symptoms, such as, fever, general fatigue, marked weakness in performance and neurological alteration (5). In Asian countries, IVLBCL predominantly accompanies a hemophagocytic syndrome know as ‘Asian-variant IVLBCL.’ due to the various, non-specific symptoms, diagnosis of IVLBCL is difficult. Usually the tumor cells do not appear predominantly in the lymph nodes, peripheral blood or organ biopsies performed for the diagnosis of IVLBCL. As a therapeutic approach to IVLBCL, chemotherapy involving the anti-CD20 antibody is usually performed, including drugs such as rituximab, cyclophosphamide, doxorubicin, vincristine and predonisolone (R-CHOP). The benefits of random skin biopsies and biopsies from senile hemangiomas for the diagnosis of IVBCL have also been reported (6,7). This report presents a case of IVLBCL diagnosed by a combination of random skin biopsies and a biopsy from a senile hemangioma to emphasize the benefits of biopsies from senile hemangiomas in the diagnosis of IVLBCL. The family of the patient provided informed consent. This study was approved by the Ethics Committee of Toyooka Hospital (Toyooka, Japan).

## Case report

An 86-year-old Japanese male without notable medical history presented to his general practitioner with continued general fatigue, loss of appetite, weight loss, fever and night sweats. Since anemia, thrombocytopenia and hypoalbuminemia were detected in blood tests, the patient was referred to Toyooka Hospital (Toyooka City, Japan). Blood examination revealed anemia, with hemoglobin, 9.4 g/dl; high lactate dehydrogenase, 282 IU/l (normal range, 106–211 IU/l); low total protein, 4.1 g/dl (normal range, 6.7–8.0 g/dl); hypoalbuminemia, 1.6 g/dl (normal range, 3.4–4.9 g/dl); high C-reactive protein, 3.38 mg/dl (normal range, 0–0.5 mg/dl); and high soluble interleukin 2 receptor, 8,250 U/ml (normal range, 145–519 U/ml), suggesting hematopoietic malignancy. However, no abnormal hematopoietic cells were detected upon examination of the complete blood cell count. As no lymph node swelling was identified on examination by whole-body computed tomography scan, intravascular lymphoma was suspected.

**Figure 1:**
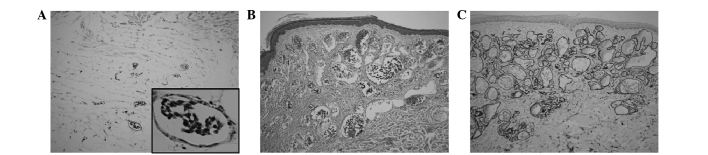
Random skin and senile hemangioma biopsies revealing atypical lymphoid cells in small blood vessels of the skin. (A) H&E staining of random skin biopsy. Small numbers of atypical lymphoid cells are visible in the small vessels of the deep dermis and subcutaneous tissue (magnification, ×10; magnification of inset photograph, ×60). (B) H&E staining of senile hemangioma. Numerous atypical lymphoid cells are present in the dilated blood vessels (magnification, ×10). (C) Anti-CD34 immunostaining of senile hemangioma (magnification, ×10). CD, cluster of differentiation; H&E, hematoxylin and eosin.

**Figure 2:**
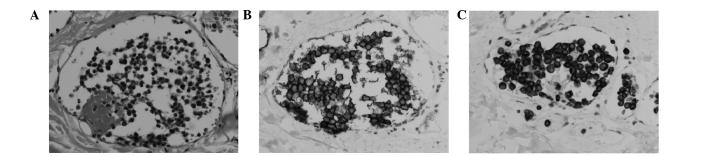
Atypical lymphoid cells in senile hemangioma expressing CD20 and Bcl-2. (A) Atypical lymphoid cells (H&E stain; magnification, ×60). (B) CD20 and (C) Bcl-2 immunostaining (magnification, ×60). CD, cluster of differentiation; H&E, hematoxylin and eosin.

Random skin biopsies were obtained from four locations: The right side of the abdomen, the left side of the abdomen, the right thigh and the left thigh. Another skin biopsy was obtained from a senile hemangioma on the left side of the abdomen. Large atypical cells with hyperchromatic nuclei and prominent nucleoli were detected in the small blood vessels in the deep dermis and the subcutaneous tissue of the specimen from the right-side abdominal skin. Atypical cells were also detected in the small blood vessels in the subcutaneous tissue in the skin biopsy specimens from the left side of the abdomen and the right thigh. However, only small numbers of these atypical lymphoid cells were present. No atypical lymphoid cells in the specimen from the left thigh were identified. However, far more atypical lymphoid cells were observable in the specimen from the senile hemangioma. In this lesion, dilated small blood vessels, which express cluster of differentiation (CD)34, were present in the superficial section of the dermis and contained numerous atypical lymphoid cells (Fig. 1). A fibrin clot was also identified in one of the vessels. Immunohistological analyses revealed that these large atypical lymphoid cells expressed CD20 and Bcl-2, but not CD3, CD5, CD10, cyclin D1, CD30 or CD34, suggesting a diagnosis of intravascular large B cell lymphoma (Fig. 2).

**Figure 3:**
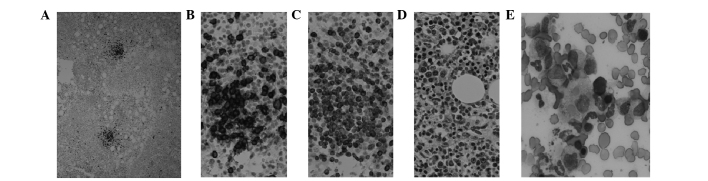
Immunohistological analyses of the bone marrow using anti-CD20 antibody. (A) Clusters of large CD20^+^ cells (magnification, ×2). High-power field reveals (B and C) Bcl-2^+^ CD20^+^ cells and (D) large cell bodies and enlarged nuclei with hematoxylin and eosin staining (magnification, ×60). (E) Bone marrow smear with May-Grünwald-Giemsa staining. Erythrophagia is visible in the bone marrow (magnification ×60). CD, cluster of differentiation.

**Figure 4:**
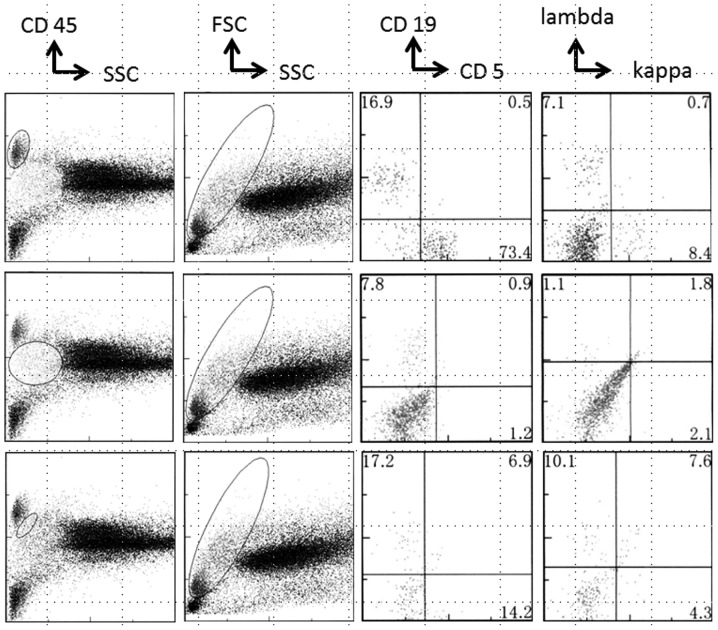
Flow cytometry revealing aberrant κ/λ chain ratio in bone marrow cells. Bone marrow cells were stained with PerCP-labeled anti-CD45 Ab, PE-labeled anti-CD19 Ab and FITC-labeled anti-CD5 Ab, or PerCP-labeled anti-CD45 Ab, PE-labeled anti-λ Ab and FITC-labeled anti-κ Ab, followed by analyses using a flow cytometer. Cells were gated by expression of CD45 and side scatter levels (left line), and the expression of CD19 and CD5 or λ and κ were then analyzed in the gated cells. CD, cluster of differentiation; FSC, forward scatter; SSC, side scatter; Ab, antibody; FITC, fluorescein isothiocyanate.

As it has been reported that the Asian variant of IVLBCL exhibits infiltration of the lymphoma cells into the bone marrow, bone marrow aspiration was also performed. As shown in Fig. 3A, in the bone marrow there was focal proliferation of CD20^+^ cells, which were immunohistologically revealed to be Bcl-2^+^, CD3^−^, CD5^−^ and CD10^−^. These cells were large with clear, enlarged nucleoli (Fig. 3B–D). Macrophages were also identified, which phagocytized erythrocytes in the bone marrow (Fig. 3E). In addition, flow cytometric analyses of the bone marrow were carried out. In the lymphocyte and blastic gates, the κ/λ chain ratio was normal (Fig. 4). However, a small population between the lymphocyte and blastic gates was identified, exhibiting lower side scatter than the monocyte gate. When the gate was set on this population, an abnormal κ/λ chain ratio was apparent, suggesting that monoclonal or oligoclonal B cells resided in the bone marrow. The immunohistological and flow cytometric analyses of the bone marrow suggested that the IVLBCL had infiltrated the bone marrow of the patient.

## Discussion

This report concerns a case of IVLBCL arising in an elderly male patient, and demonstrates the benefits of the combination of random skin biopsies and a biopsy of senile hemangioma for diagnosing IVLBCL.

IVLBCL is a rare lymphoma, characterized by the selective growth of lymphoma cells within the lumina of the blood vessels (5). It has been reported that IVLBCL arises in the elderly, and that fever and hemophagocytic syndrome are common in the Asian variant of IVLBCL (5). In the present case, the patient was 86-years-old and presented with weight loss, fever and night sweats (B symptoms). Examination of the bone marrow revealed the presence of hemophagocytic syndrome. These results suggest that this case is consistent with Asian-variant IVLBCL.

Various levels of CD5 and CD10 expression in IVLBCL have been reported (4). It has been reported that CD5^+^ and CD10^+^ lymphoma cells are present in 38 and 13% of IVLBCL cases, respectively. CD10^−^ IVLBCL has been categorized as a non-germinal-center lymphoma in Asian patients (4). In the present report, CD5 and CD10 were negative, consistent with the dominant type of IVLBCL in Asia. Therefore, on the basis of symptoms and laboratory results, this case would be considered an example of Asian-variant IVLBCL.

It has been suggested that lymphoma cells reside in the small vessels in various organs in IVLBCL (8). Therefore, various biopsy sites have been reported in the diagnosis of IVLBCL. These include the bone marrow, skin, prostate, testicle, adrenal gland, brain, liver, kidney and lacrimal gland (4,5,9–13). It has also been reported in Asian cohorts that the optimal diagnostic site is the bone marrow (4). The usefulness of random skin biopsies in the diagnosis of IVLBCL has also been reported (6,14–17). Skin biopsies can be carried out easily, and random skin biopsies have been reported to be useful in the diagnosis of not only European IVLBCL patients, in whom the cutaneous variant is prevalent, but also Asian IVLBCL patients, in whom the cutaneous variant is rare (5,18). It has also been reported that the greater the number of random skin biopsies carried out, the more accurate the diagnosis of IVLBCL (15). However, some patients test negative upon random skin biopsy (5). In the present case, lymphoma cells were detectable in samples from random skin biopsies, but there were few lymphoma cells in the sample blood vessels. Lymphoma cells mainly resided in the blood vessels of the deep dermis or subcutaneous adipose tissue; therefore, if careful observations had not been made, or had the skin biopsies not contained deeper layers of the skin, the lymphoma cells may have been missed.

The benefit of biopsies of senile hemangiomas for the diagnosis of IVLBCL has been previously reported (1,19). In the reported cases, lymphoma cells were detected only in the blood vessels of the senile hemangioma, while random skin biopsy specimens from apparently normal skin failed to reveal any lymphoma cells. In the present case, numerous lymphoma cells were identified in the senile hemangioma. As senile hemangiomas are usually present in the superficial dermis, biopsies can be easily obtained. Therefore, if IVLBCL is suspected and the patient has a senile hemangioma, a combination of random skin biopsies and biopsy of the senile hemangioma should be carried out in order to make a firm diagnosis.
